# Multiple Micronodular Pneumocyte Hyperplasia (MMPH): A Less-Known Presentation of Tuberous Sclerosis Complex Related Lung Disease

**DOI:** 10.5334/jbsr.3111

**Published:** 2023-04-13

**Authors:** Matthias Lembrechts, Adriana Dubbeldam

**Affiliations:** 1 UZ Leuven, BE

**Keywords:** Multiple micronodular pneumocyte hyperplasia, MMPH, Tuberous sclerosis complex, TSC, ground glass nodule

## Abstract

**Teaching Point:** Multiple micronodular pneumocyte hyperplasia (MMPH) is a less-known presentation of tuberous sclerosis complex (TSC) associated lung disease that usually requires no treatment or follow-up imaging.

## Introduction

Tuberous sclerosis complex (TSC) is a genetic systemic disorder characterized by the formation of widespread hamartomas and other dysplastic lesions in various organs. The typically affected organs are the brain, kidneys, lungs, skin, and heart.

Pulmonary involvement is frequent in patients with TSC, most typically lymphangioleiomyomatosis (LAM). LAM is characterized by multiple cystic lung lesions, which can lead to pneumothorax or chylous pleural collection and ultimately can cause progressive pulmonary failure. Multiple micronodular pneumocyte hyperplasia (MMPH) is a second and lesser-known presentation of lung disease associated with TSC. Early diagnosis of MMPH on computed tomography is critical to prevent unnecessary treatment or follow-up, since MMPH is not progressive and is asymptomatic.

In this case report, we will discuss a patient with TSC and signs of MMPH on a screening computed tomography scan of the chest.

## Case History

We present a case of a 37-year-old woman with a history of tuberous sclerosis without any respiratory complaints.

**Figure 1:**
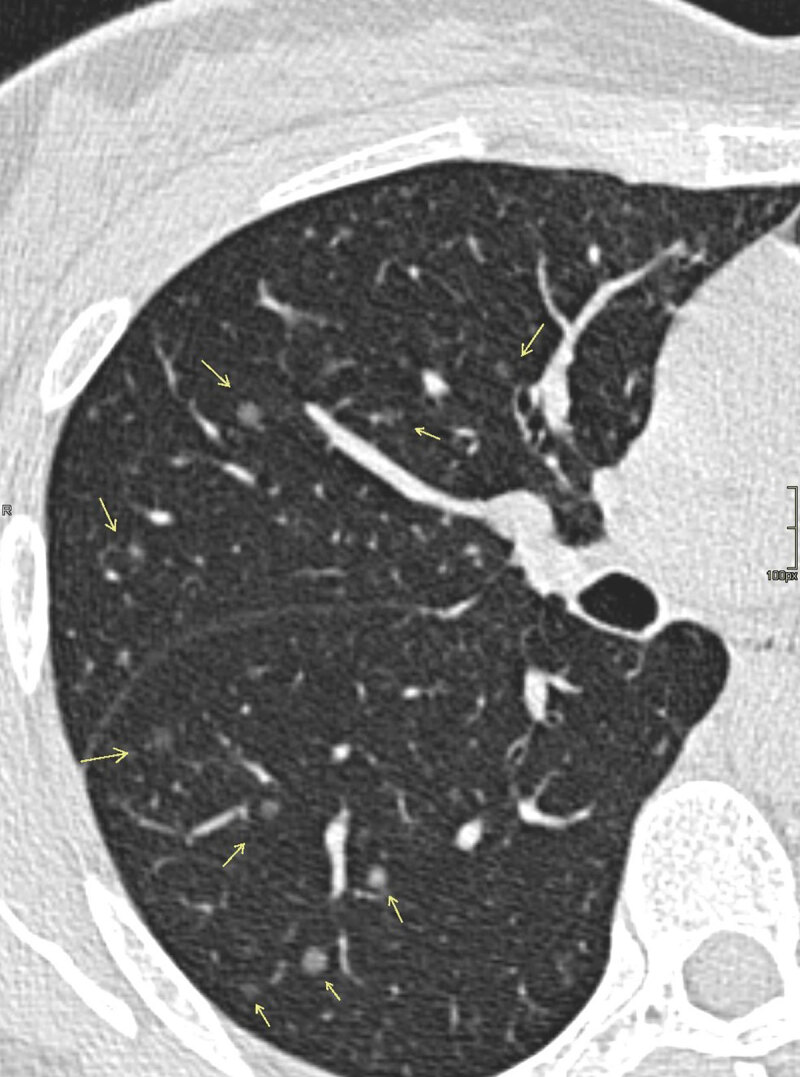


**Figure 2:**
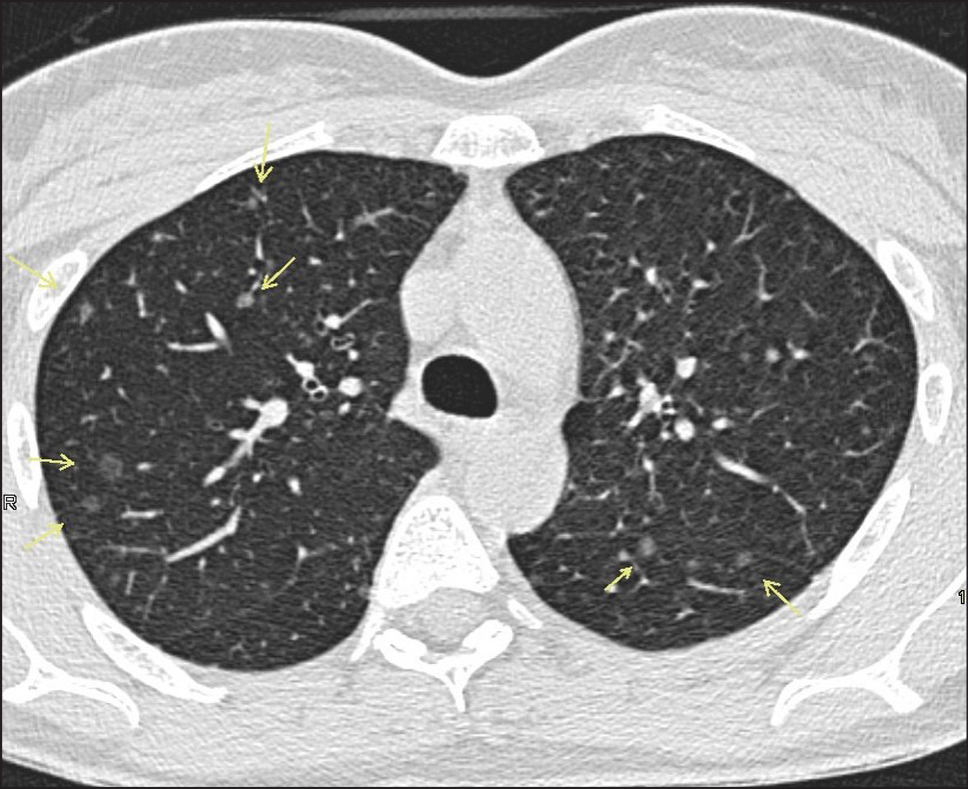


A computed tomography scan was ordered to examine lung involvement secondary to TSC. Multiple scattered nodules, both solid and ground glass, were noted in a random distribution pattern (Figures 1, 2). The maximum nodule diameter measured up to 10 mm.

## Comments

Lymphangioleiomyomatosis (LAM) and multifocal micronodular pneumocyte hyperplasia (MMPH) are both classic manifestations of lung involvement in TSC. The prevalence of LAM is estimated at around 40% of female patients with TSC and increases with age up to around 80% prevalence in patients above 40 years old.

LAM presents typically with progressive cystic lesions in the lungs which can cause recurrent pneumothorax, chylous pleural collection and can lead to progressive pulmonary failure. A lesser-known presentation of pulmonary involvement of TSC is multiple micronodular pneumocyte hyperplasia (MMPH). MMPH is characterized by the proliferation of type II pneumocytes along the alveolar septa combined with an increase in alveolar macrophages, fibrotic interstitial thickening, and possible lymphocytic infiltration [1]. MMPH affects men and women equally. Both MMPH and LAM can occur together, as the pathophysiology of both the diseases is different.

**Figure 3:**
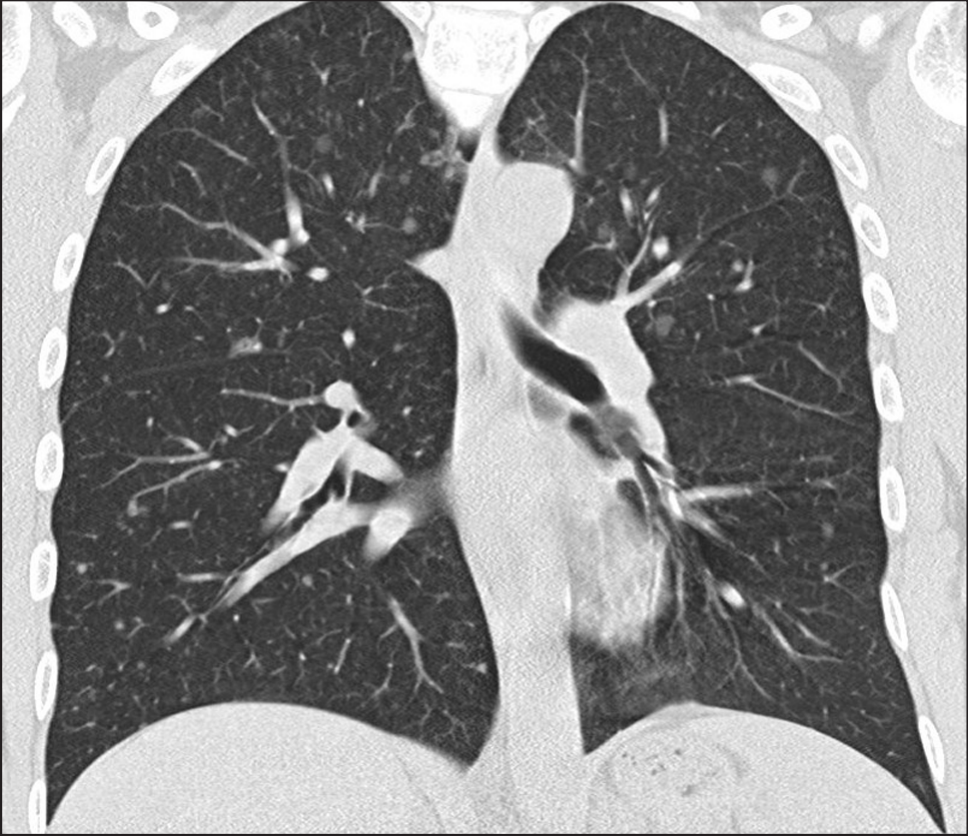


MMPH typically presents multiple pure ground glass nodules in a random distribution pattern (Figures 1, 2, 3). These ground glass opacities correlate to the pneumocyte proliferation and infiltration of macrophages in the alveolar septa. Patients are classically asymptomatic and lesions are not progressive, which is why treatment or follow-up is typically not necessary.

## Conclusion

Multiple micronodular pneumocyte hyperplasia is a less-known presentation of TSC associated lung disease compared to lymphangioleiomyomatosis. It presents as multiple pure ground glass nodules with a random distribution pattern on computed tomography. Patients with MMPH are typically asymptomatic and lung lesions are not progressive. Treatment or follow-up imaging are therefore unnecessary.
